# Choosing a women’s health career

**DOI:** 10.1186/s12909-018-1362-4

**Published:** 2018-11-06

**Authors:** Isabel C. Green, Alessandra J. Ainsworth, Julia Riddle, Dawn M. Finnie, Betty Chou

**Affiliations:** 1 Department of Obstetrics and Gynecology, Mayo Clinic, 200 First St SW, Rochester, MN 55905 USA; 2 Robert D. and Patricia E. Kern Center for Science of Health Care Delivery, Mayo Clinic, Rochester, MN USA; 3 Department of Psychiatry, Johns Hopkins Hospital, Baltimore, MD USA; 4 Department of Gynecology and Obstetrics, Johns Hopkins Hospital, Baltimore, MD USA

**Keywords:** Medical student, Obstetrics and gynecology, Recruitment

## Abstract

**Background:**

In 2005, in response to a decline in residency applications in obstetrics and gynecology (OB GYN), the American College of Obstetrics and Gynecology Presidential Task Force outlined strategies for attracting medical students to OB GYN. Application rates have increased since then, but little is known about which interventions are effective. We aimed to identify modifiable and nonmodifiable variables that may contribute to students choosing OB GYN for their careers; this information could be used to inform curriculum design, faculty development, and innovative exposures to women’s health.

**Methods:**

This qualitative study received institutional review board approval. Eligible participants were students who applied or recently matched into OB GYN residency programs from the class of 2014–2016 at our institution. Students were interviewed with open-ended questions and a Likert-type survey. Thematic analysis was performed.

**Results:**

Ten qualitative interviews were completed and analyzed. Intrinsic themes such as the potential for a meaningful job in women’s health, advocacy for women, or empowerment of women were identified as factors contributing to participant career choice. Extrinsic themes such as positive impressions during the clinical clerkship and welcoming teams were also identified. Most students indicated that the clerkship was the most influential experience.

**Conclusions:**

Participants identified important events, including some that even preceded medical school that guided them toward OB GYN. The data guide us to consider the importance of emphasizing the unique combination of characteristics in OB GYN and improving the learning environment in the clerkship as a way to encourage student recruitment.

**Electronic supplementary material:**

The online version of this article (10.1186/s12909-018-1362-4) contains supplementary material, which is available to authorized users.

## Introduction

In the early 2000s, the number of students entering the field of obstetrics and gynecology (OB GYN) in the United States was decreasing [1]. Researchers investigating student experiences on the clinical clerkship identified areas of concern and potential improvement [2–5]. In response to this decline in applicants, in 2005, the American College of Obstetrics and Gynecology (ACOG) outlined strategies for recruiting medical students. These suggestions included improving the medical student clerkship experience and creating opportunities for early exposure to OB GYN [1]. At our institution, multiple interventions were launched in response. One intervention was the implementation of a preclinical anatomy course, which targeted early exposure. In this course, OB GYN residents and faculty were brought into the anatomy laboratory to supplement teaching with a team-based learning session and resident-led prosection reviews. Similar strategies to improve early exposure have been implemented in general surgery, with some success [6–11].

Since 2005, trends in recruitment to OB GYN have improved. In the 2017 Match, all residency slots were filled (82% filled by US graduates) [12]. However, the reasons for this improvement are not well known. A better understanding of the impact of deliberate interventions is needed to strengthen their effect, to allow for judicious use of resources, including faculty time, and to continue recruitment success. Prior studies have explored this question, primarily with survey-based instruments [4, 13, 14]. Nevertheless, a qualitative understanding is needed to fully capture what is and is not affecting the decision to go into OB GYN. The primary aim of this study was to identify factors that draw students to the field of OB GYN; specifically, this investigation evaluated characteristics specific to the specialty, as well as modifiable experiences such as the clerkship and interventions aimed at early exposure.

## Materials and methods

For this study, we used semistructured interviews to identify factors that influenced medical students’ career choice. The Johns Hopkins Review Board approved the study. Informed consent was obtained from study participants.

### Setting

The research was conducted at an academic medical center in the United States. All medical students are required to complete a 4-week pre-clinical didactics based course in reproductive medicine in their 2nd year, and an 8-week clinical clerkship in OB GYN during their 3rd year or early 4th year of their four-year medical school education. In the fall of their 4th year, students select and apply to a desired training program (medicine, surgery, OB GYN, etc.) in a process termed the Residency Match.

### Participants

Purposive sampling was used to identify key informants with recent and relevant experience. Students who had attended the Johns Hopkins School of Medicine and matched into OB GYN residency positions from 2014 through 2016 were eligible for the study. These individuals had been students when a series of cross-curricular interventions, designed to promote recruitment to OB GYN, had been implemented. They were invited to participate via email, and study recruitment was timed to occur after the 2016 Residency Match. Participants were offered a $50 gift card as remuneration.

### Interview guide

We developed a semistructured interview guide (Additional file 1). Interview questions were designed as part of a mixed-methods research course in the Master of Health Profession Education graduate program by one author (I.C.G.) using the following reference [15]. Open-ended questions were designed to explore participant’s experiences that influenced their decision to pursue OB GYN. In addition, Likert-type questions were included to specifically assess the perceived level of influence of discrete institutional curriculum components on career choice. The decision to include Likert-type questions was to address the potential impact of specific resources employed at our institution with the intention of improving the student experience and recruitment. Items were scored on a 0–5 scale (0 = not applicable, did not attend; 1 = extremely negative; 2 = negative; 3 = neutral; 4 = positive; 5 = extremely positive). These were intentionally placed at the end of the interview guide to conclude the interview and not bias the responses to the open ended questions. The curriculum components and descriptions are included in Table 1. The guide was pilot-tested during a peer-review process and questions were subsequently revised and focused.

**Table 1:** Curriculum components at study institution

Women’s health interest group: Student interest group, focused on preclinical years	
Birthing experience: Opportunity for preclinical students to attend a vaginal delivery and shadow a patient	
“What does an OB GYN do?” event: Evening workshop for preclinical students to learn more about OB GYN practice and careers through simulation stations and small-group sessions	
First-year gross anatomy course: Pelvic anatomy session with OB GYN faculty and residents	
Genes-to-society reproduction course: Preclinical pathophysiology course on the reproductive system	
Mentorship with residents and/or OB GYN faculty	
PRECEDE (*PRE-C*lerkship *ED*ucational *E*xperience): A simulation and didactic orientation curriculum during the OB GYN clerkship	
Clinical responsibility during the OB GYN clerkship	
Contact with faculty and residents during the OB GYN clerkship	
Subinternship in OB GYN	
Electives in OB GYN	

Abbreviation: *OB GYN* obstetrics and gynecology

### Interview methods

One-on-one interviews were conducted by telephone from March 2016 through September 2016. A trained (J.R.) female medical student familiar with the OB GYN residency program, but independent from the program, served as the interviewer. Interviews were audio recorded, transcribed verbatim by an administrative assistant, and deidentified prior to analysis.

### Analysis

Thematic analysis, as described by Braun and Clarke, was conducted [16]. Three researchers (I.C.G., A.J.A., and D.M.F.) analyzed the data, led by one analyst (D.M.F.) from the Qualitative Research Unit within the Mayo Clinic Robert D. and Patricia E. Kern Center for the Science of Health Care Delivery, an individual trained in qualitative methodology. Descriptive statistics were utilized for the Likert-type questions. Participant comments generated during the Likert-type questions in the interview were included with the qualitative data for thematic analysis.

The six phases of thematic analysis include: 1) familiarizing yourself with the data, 2) generating initial codes, 3) search for themes, 4) reviewing themes, 5) defining and naming themes, and 6) producing the report [16]. We began by independently reading through transcripts, taking notes on ideas and themes. We used an open coding process to generate initial codes. The coding scheme was then applied to all transcripts. As codes were repeated across interview transcripts, emerging themes were identified and refined throughout the analysis process. We met to confer until consensus was reached, adjudicating differences through discussion. NVivo software (QSR International Pty Ltd.) was used to manage and query the data. The reporting of the study was in accordance with the COREQ (consolidated criteria for reporting qualitative research) statement [17].

## Results

Thirteen graduates were eligible for inclusion; 3 declined because of scheduling difficulties and 10 interviews were completed (response rate, 77%). Seven interviewees were women. One interview was repeated because of poor recording quality and concluded before answering the Likert questions.

Seventeen unique themes were identified. The themes were categorized into experiences or qualities intrinsic or extrinsic to the individual or to the field of OB GYN. The themes are presented in Table 2 with representative quotations. Intrinsic themes reflected both qualities of the field of OB GYN and personal characteristics of the participant. Extrinsic themes reflected the educational environment and highlighted the role of interactions and experiences that motivated participants towards selecting OB GYN.

**Table 2:** Intrinsic and extrinsic motivators for selecting obstetrics and gynecology

Theme	Participant Quote
Intrinsic	
Advocacy and empowerment	“OB GYN had elements of advocacy that I had always wanted and always been really important to me”
Personality and characteristics	“I feel like I have a good relationship with people when it comes to uncomfortable topics and making them comfortable. I think this will help me in OB/GYN in uncomfortable situations for people to have that experience in patient care. I just feel very natural in talking with women.”
Variability of practice	“I really liked how with OB/GYN you can go from all aspects of patient care from happy times to announcing birth to hardships and time of death. You really get all aspects of continuity of care from a primary provider, but additionally as well with more hands-on experience in terms of surgery and C-sections.”
Content	“I remember being really enamored by the subject material, the anatomy and just the physiology”
Meaning	“I love being able to see the direct impact you can have. In a lot of settings, you’re the only providers that they see, and you have the opportunity to make a huge difference.”
Relationships	“So, I kind of like that with patients that you could establish a relationship and talk about what’s very personal and important to them, and something that they really wouldn’t discuss or feel safe outside of a clinical setting without having established a relationship between a physician and patient.”
Intensity	“The fact that things can go terribly wrong, and the potential to experience one of the worst days of your life and to be prepared to be present and helpful in that scenario as well. It’s really just a unique opportunity”
Society and policy	“A lot of crap laws have been passed in various states, and I am a firm proponent of increasing choice and trying to limit the anti-choice measures that have been so popular in conservative states. Those things outside of school have increased my desire to go into the field”
Immediacy	“I enjoy working with my hands and really fixing something. I really needed immediate gratification and you definitely get that with delivery”
Extrinsic	
Role models and mentors	“She was just a phenomenal physician and teacher, and that was a really nice example for someone I would like to model my residency path after.”
Teams	“Really the one time in medical school that I felt I was part of the team… I had a valuable role and I wasn’t as much just an observer but I was contributing to the work of the day and actually helping with patients.”
Community	“Seeing how well I got along with [students applying into OB GYN] and that we had very similar values and interest in medicine and what we wanted to do.”
Inclusion	“It was a welcoming and inviting comment, and it made me feel included and part of the team.”
Exposure	“Without contact with faculty, there is no way that I could have done that visualization. I could not have seen myself in their shoes.”
Teachers	“I think [my teacher] did a really good job of making it exciting and appealing.”
Organization	“[The course] was very well shaped, well thought out, and extremely coordinated. It really flowed, it made sense…That really got my mind around reproductive science and women’s health when I really hadn’t considered it as a career before.”
Value of education	“The residents felt they were well supported and that people care about their education, which is different than you felt in some other [fields].”

Abbreviation: *OB GYN* obstetrics and gynecology

### Intrinsic themes

#### Advocacy and empowerment

All participants reflected on the role of advocacy and empowerment of women as a motivator. The possibility of “finding ways to empower women and [others]” in their future careers was clearly appealing, and empowerment was discussed at the individual and population levels. Opportunities for empowerment were described generally for women’s rights and a potential for empowerment that could exist on a daily, patient-based level.

All the new laws against women and their reproductive rights has made [OB GYN] even more appealing, and getting involved in policy or advocating for health and the opportunities that come with being a professional women’s health physician. (ID#01, male)

One thing that really made me more proud and want to go into OB GYN is definitely the whole controversy surrounding abortion and Planned Parenthood that has been going on. It has made me realize there is still a big fight that needs to happen for women’s rights. I think OB GYNs have a very crucial part in that. That really helped confirm that’s what I want to do, because I feel there’s still so much that needs to be done in the world. (ID#04, female)

One participant highlighted the difference between a surgical career in OB GYN vs one in general surgery by relating it to personal fulfillment from advocacy.

The biggest piece of it was feeling like surgery in and of itself would not be personally fulfilling for me, and that OB GYN had elements of advocacy that I had always wanted and always been really important to me, and realizing that I could make a career out of it instead of just doing advocacy as a side project made me realize this was the right path. (ID#03, female)

Themes of empowerment also extended to colleagues and peers. One participant reflected on her experience in an all-women student organization, saying it was the “first time I had worked on a team that was exclusively female and really talked about issues regarding feminism and being a woman in medicine,” which affirmed her interest in OB GYN.

#### Personality and characteristics

All participants commented that OB GYN was a “good fit” with their personality or personal characteristics. A few participants elaborated further by describing their ability to be good listeners, create a trusting space for discussion of sensitive health issues, or handle uncomfortable questions and issues from peers, family, or patients.

#### Variability of practice

All participants commented that the variability of practice in OB GYN was appealing, and a few described the possibility of indecision and delaying their career choice as positive attributes. Within OB GYN, they could further decide whether to have a primarily surgical or medical practice, to focus on research or clinical care, and to explore public health or clinical practice. Some participants appreciated the multitude of options (subspecialities) within an OB GYN career, whereas others appreciated the potential for variety (often the combination of surgery and medical care) in their future practice.

#### Content

Participants commented on their interest in the content of OB GYN indicating that these subjects often resonated more compared with the content of other specialties. They described an affinity for the subject when they encountered it in the preclinical and clinical curriculum. Two participants commented on content outside the curriculum. One felt a “pull” and interest in OB GYN on the basis of national trends in the field such as debates surrounding the ethics of genetics and preimplantation screening. Another felt that her decision to enter OB GYN was validated after attending medical conferences in OB GYN and “realizing I am legitimately interested in what they have to say.”

#### Meaning

The possibility of being part of a meaningful event in a patient’s life, often cited as the birth of a child, was an appealing aspect of OB GYN. Meaning was also derived from the desire to directly affect patients, including those seeking care during vulnerable moments.

#### Additional intrinsic themes

Other intrinsic themes included relationships, intensity, society and policy, and immediacy. Participants referenced an affinity for the patient population and opportunities to forge short- and long-term relationships with patients. For a few participants, the intensity of some moments in OB GYN was attractive. For 2 participants, intensity was identified in workflow and pace, whereas the others identified intensity in a clinical context such as disclosing the diagnosis of a lethal fetal anomaly. Some participants believed that the field of OB GYN was intertwined with society and public policy because of the nature of women’s health issues, and this link was appealing. Immediacy (or immediate gratification) in surgery or deliveries was also referenced.

### Extrinsic themes

#### Role models and mentors

Participants described the positive impact of role models in OB GYN. Many described residents and faculty that exemplified the type of physician that they hoped to become.

The way she treated patients and how dedicated she was demonstrated exactly who I wanted to be. (ID#07, female)

Others were motivated by seeing an example of a career trajectory that they could envision as their own.

I met some amazing faculty who I could see myself wanting their career and doing what they were doing. That was definitely encouraging to see that there are people out there who have already done and who have paved the way for something I would be thinking about doing. (ID#01, male)

Seeing her and the example that she was setting in the academic field as an OB—there aren’t many OB/GYNs that are high up like that in the education world, but [are] still an OB GYN clinically….[It] is a great thing to see an example of someone I could work for. (ID#05, female)

Many participants described important relationships with mentors outside of OB GYN. Effective mentors provided guidance, avoided passing judgment or pressuring them to enter a particular field, and gave the participant “space to explore my career.” Two participants drew inspiration from their mentors’ passion for their own careers, inspiring one to want “to be as excited about my job in the future.”

#### Teams, community, and inclusion

The clinical clerkship and educational environment offered opportunities for positive and negative influences. One participant described contradictory experiences with different resident teams.

One of the first residents that I worked with on my first week was just so welcoming and kind and so wonderful to me. [She] and some others made me so excited about working with people like that in the field. There were some [residents] who I did not like as much … who I felt would be difficult to be a co-resident with. (ID#03, female)

However, most participants reflected positively on the sense of inclusion, community, and teamwork, and remarked on inviting moments or comments.

I was really impressed at how much clinical responsibility we got in terms of doing notes, following patients, being in the OR, and rounding on our patients in the morning. I felt like we got continuity and we were part of the team, unlike some other services. It was really positive. (ID#10, female)

[I was] impressed with the way that the residents on my team handled their responsibilities and interacted with patients and were thoughtful about my role as a medical student. Had I not felt sort of invited, and at that time I had some concerns about being a guy and being interested in OB GYN, and had those be confirmed through these nonexplicit experiences, then I may not have chosen this specialty. (ID#02, male)

#### Additional extrinsic themes

Exposure, teachers, organization of educational encounters, and evidence of the value placed on student education were other extrinsic themes. Many appreciated opportunities to learn about the breadth of the field through exposure to different specialties or different practice types. For some, this exposure occurred in selectives during the clerkships, whereas others described exposure to faculty and programs outside the institution such as global health initiatives. High-quality educators, organized courses, and a sense of “value” of education witnessed in the OB GYN department were highlighted.

### Response to specific program interventions at the study institution

**Fig. 1:**
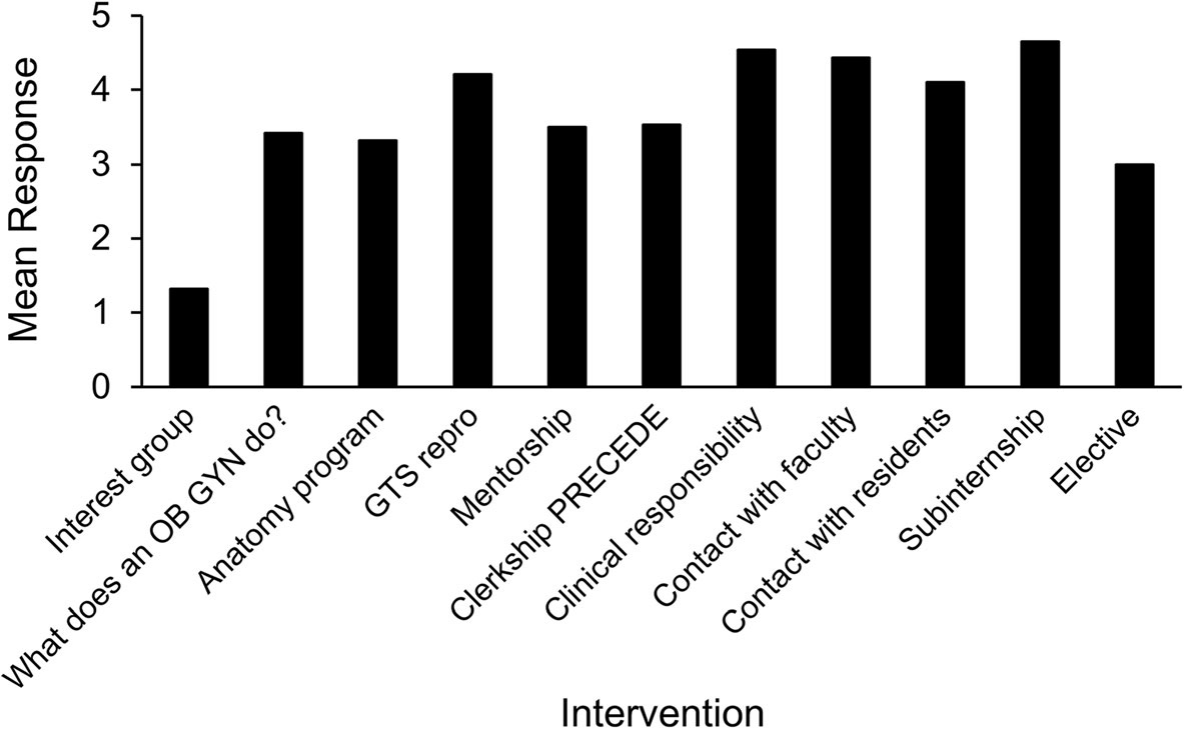
Survey on Program Interventions. Experiences are listed chronologically, from left to right. Potential responses to the Likert-type questions ranged from 1 to 5. GTS repro indicates genes-to-society reproduction course; PRECEDE, PRE-Clerkship EDucational Experience

The results of the Likert-type questions are listed in the Fig 1. Interventions or experiences later in the course of education had greater self-reported impact than those in the preclinical years, with the exception of the second-year preclinical reproductive course (mean [SD], 4.22 [0.83]; range, 0–5). Some participants stated they would have given a higher score to contact with residents and the clerkship experience, except that some negative encounters had detracted from the positive ones and thus lowered their overall rating. Many early experiences, such as involvement in an interest group and OB GYN career night, were not consistently utilized or attended. The “birthing experience” (opportunity for preclinical students to attend a vaginal delivery and shadow a patient) was ultimately excluded from the analysis. During the first 4 interviews, the interviewer failed to clarify that this question was referring to the formal activity for preclinical students; participant answers described the childbirth experience in general.

Most participants identified the clerkship as the first time they perceived a true interest in the field. Upon reflection, some identified an interest in women’s health that preceded the clerkship, but the clerkship still was instrumental in directing them toward an OB GYN career. A few participants also described deciding on OB GYN after completing other clerkships. These non − OB GYN rotations helped participants solidify their interest in OB GYN by 1) identifying aspects of medicine that they found most fulfilling and 2) highlighting other aspects that they did not enjoy.

### Barriers

A negative reputation for the field was a common barrier, but it was one that participants described overcoming and being satisfied with their decision. Most participants cited work-life balance and the potential for long hours as possible barriers, but these ultimately did not deter them from their choice. Liability was a common concern voiced by parents of the participants.

## Discussion

Opportunities for advocacy, meaning in work, and establishing valuable relationships with patients were intrinsic factors central to the decision to become an OB GYN. This field commonly intersects with vulnerable patients, sensitive medical issues, and the chance for long-term patient care, and it is also influenced by national and state policies. Each of these characteristics could be duplicated in other fields—for example, primary care certainly engages in long-term relationships with patients, and trauma surgery and oncology provide opportunities for life-saving interventions. However, participants seem to have honed in on a unique combination of these factors in OB GYN that contributed to finding meaning in the work.

The role of advocacy in job satisfaction is well described in the nursing literature, with advocacy on the behalf of an individual patient being considered inherently rewarding [18]. However, public advocacy represents engaging in social, economic, educational, and political changes and reflects the type of advocacy described in this study [19]. Physicians involved in continued public advocacy describe “deep-rooted drives and passions, and the potential to increase and perpetuate engagement” [20].

In general, physicians who identify their practice as a true calling are motivated by an ability to promote a greater good and are more likely to derive meaning from their work than physicians who identify their career as a job [21]. This sense of meaning is protective against physician burnout and is associated with career and life satisfaction [21, 22]. The intrinsic motivators of advocacy and meaning identified in this study represent not only qualities that draw students to the field but traits that may have a significant impact on career satisfaction and continued work in medicine [23]. Although most participants also cited work-life balance as a concern (but one that ultimately did not prevent them from choosing OB GYN), intrinsic motivation may be a key area for future exploration.

The clinical rotations (i.e., clerkship and subinternship) were described as very influential. The clinical rotations had the most impact and seemed to be the centerpiece for recruitment. Clerkships have a marked influence on specialty selection in many fields and specifically in OB GYN [5, 24, 25]. However, the OB GYN clerkship and educational environment create opportunities for both positive and negative influence [3, 4], which was confirmed in our study.

Participants reflected positively on the sense of inclusion, teamwork, and community that defined their clerkship. Study participants recalled specific moments—“it was a welcoming and inviting comment, it made me feel included and part of the team” and general feelings—“it just felt like I had found a group of people that I resonated with.” These comments reflected active moments of inclusion, such as an invitation to participate in the operating room or in patient evaluations, and an innate sense of shared purpose and camaraderie. Feelings of teamwork and having opportunities for active involvement have been cited as significant features of satisfaction and interest in surgical subspecialities [26, 27]. Teamwork and inclusion remain important factors in job satisfaction in medicine in general, with studies identifying increased or improved job satisfaction from cohesive working environments in health care [28–30]. Lack of inclusion or overt exclusion has also been cited as a significant cause of dissatisfaction in OB GYN clerkships [2]. Because of patient privacy and advocacy concerns, students often are not actively involved in patient care [2]. Our in-depth interviews offer insight into successful methods of inclusion, which could be as simple as an inviting comment. Most participants highlighted a sense of being part of a team, even when they were not performing key procedures.

Limitations of this study include the homogeneity of applicants reflecting experiences at a single institution. Also, the cumulative effect of early interventions is difficult to assess when evaluating later experiences because of recall bias. However, the limitations are offset by the quality of the in-depth interviews, as evidenced by the detailed themes explored. Ten of the 13 eligible applicants (77%) were interviewed, representing the majority of applicants from the years of instituted curricular reform. Women were represented in a similar fashion to national applications, with approximately 70 to 80% of residency slots being filled by females each year [12]. Although our participants were from a single institution, the high response rate strengthens our findings. These data can serve as a pilot study that could be repeated regionally or nationally to obtain a more generalizable sample.

### Implications for practice

These interviews provide new and valuable data that can inform curriculum and program reform. The data guide us to consider 1) improvements in the learning environment; 2) the importance of emphasizing the unique combination of characteristics in OB GYN; and 3) maintaining focus on the clerkship. In the early years, the learning environment can be improved through the organized delivery of preclinical content, with consideration to adult learning principles.

Even more important, however, is the learning environment during the clerkship. Our data support interventions in team building, teaching the teachers’ curricula, and fostering role models and mentors among residents and faculty. Clinical teams are central to student recruitment because they provide the immediate learning environment for the clerkship. Team-building exercises can strengthen communication and interpersonal relationships, allowing the team to work as a cohesive group [31]. Effective faculty educators are key, as are residents who serve as near-peer teachers. Teaching the teachers’ curricula enhances resident self-confidence in teaching and improves teaching behaviors, although existing studies are limited to showing only the short-term impact of these curricula on learning outcomes [32]. A teaching curriculum in OB GYN should include coaching for residents to identify more subtle ways to include students in the team. Inclusive comments may be as powerful as active involvement in a procedure. Role models within and outside of OB GYN were essential to the evolution of career choice, and faculty development in this regard should be encouraged.

Participants honed in on a unique combination of intrinsic features of OB GYN that should be advertised to students. If opportunities for early exposure are provided, they should include aspects of advocacy, policy, the chance for meaningful relationships, and the variety of practice. These aspects should not be described in isolation—rather, they should be presented as a combination of features that distinguishes OB GYN from other fields. Finally, efforts should prioritize optimizing the clerkship. Specific interventions reviewed in this study seemed to have a greater effect with increasing stage in medical education and suggest that resources may be best spent on interventions performed during the clinical years. This need to refocus is evident when considering the preclinical anatomy course, a high-effort intervention that ultimately seemed to have little impact on interest in the field, based on students’ descriptions.

Due to the small number of participants, we were unable to draw conclusions in reference to the influence of gender on student perspectives. Interestingly, some female participants commented on the community of women in the field as appealing, while male participants mentioned the importance of feeling included in their reflections on their clerkship experiences. As the demographics of residency programs evolve in the US, this is another area for further inquiry [33].

## Conclusion

Students who chose to specialize in OB GYN identified multiple themes that drew them to the field. Although some themes seemed intrinsic to the specialty and its providers, others reflected learner experiences and interactions. Innate characteristics such as the opportunity for advocacy or close relationships with patients may be less amenable to direct interventions, but they could be highlighted during efforts toward early exposure. Themes that are extrinsic to the field itself offer opportunities for interventions to increase student interest. Finally, although the early years are helpful for increasing awareness of the field, the clerkship remains the most important recruitment tool.

## Additional file

Additional file 1: Interview Guide; interview script with questions and prompts. (DOCX 17 kb)

## Abbreviations

ACOG: American College of Obstetrics and Gynecology
COREQ: Consolidated criteria for reporting qualitative research
OB GYN: Obstetrics and gynecology
