# Supplementary material for: Choosing a women’s health career
Source: BMC Med Educ. 2018 Nov 6;18:251. doi: 10.1186/s12909-018-1362-4 (PMC6220517; doi:10.1186/s12909-018-1362-4)
Supplement: Supplementary file 1 — Interview Guide; interview script with questions and prompts. (DOCX 17 kb) [file 12909_2018_1362_MOESM1_ESM.docx]

Example Resident Survey

Introduction:

Thank you for taking the time to speak with me about your decision to choose OB GYN for your career.

I am going to start with a few open ended questions, and then end with a survey. This should take no longer than 20 min. Your completion of this survey will serve as your consent to be in this research study.

*Interviewer will start with the numbered question, and use sub questions to guide further discussion. If participant mentions experiences outlined in a sub question, the interviewer should still repeat the sub question, but can use the phrasing in italics (which other experiences…) The answers will be recorded, transcribed and then coded for analysis. The interviewer may take notes during the interview to help guide follow up questions or ask for clarifications and to keep track of the interview progress.*

1. When did you first know that you wanted to be an OB GYN?
2. Why did you choose OB GYN for your career?
   1. Which experiences (*or which other experiences)* **PRIOR** to medical school impacted your decision?
   2. Which experiences *(or which other experiences)* **DURING** medical school and part of the **EXPLICIT** curriculum (years 1-4) impacted your decision?
   3. Which experiences *(or which other experiences)* **DURING** medical school and **NOT** part of the explicit curriculum (years 1-4) impacted your decision?
   4. Which experiences *(or which other experiences)* **OUTSIDE** of medical school but during your medical school years impacted your decision?
3. What role did mentorship, formal or informal, play in your decision?
4. What was the reaction of your classmates to your decision in OB GYN?
5. What was the reaction of your family to your decision in OB GYN?
6. Did anything deter you from OB GYN, or contribute to some doubt?
7. Are you happy with your decision now?
   1. Do you have any regrets?
   2. Which experiences (good or bad) have changed your degree of satisfaction with your choice?

Survey:

N/A – did not participate or attend

Extremely negative – made you question your decision

Negative

Neutral/ No impact

Positive

Extremely positive – is a highlight for your decision

1. Involvement in the Women’s Health Interest Group
2. The Birthing Experience
3. What Does an OB GYN Do workshop
4. Anatomy Program (Year 1 lecture, TBL, resident prosections)
5. GTS Reproduction Course
6. Mentorship with OB GYN Resident or Faculty
7. Clerkship Simulation (PRECEDE)
8. Clinical Responsibility on the Clerkship
9. Contact with Faculty on the Clerkship
10. Contact with Residents on the Clerkship
11. Sub-Internship (which Sub-Internship did you perform)
12. Elective (which elective did you perform)
    1. Elective ________
    2. Elective ________
    3. Elective ________
